# The relationship between reduced choroidal thickness due to high plasma asymmetrical dimethylarginine level and increased severity of diabetic retinopathy

**DOI:** 10.5935/0004-2749.20230007

**Published:** 2023

**Authors:** Umut Dağ, Mehtap Çağlayan, Mehmet Fuat Alakuş, Hasan Öncül

**Affiliations:** 1 Department of Ophthalmology, University of Health Sciences Diyarbakir Gazi Yasargil Training and Research Hospital, Diyarbakir, Turkey

**Keywords:** Diabetes Mellitus, Type 2, Diabetic Retinopathy, Choroid, Fovea Centralis, Nitric Oxide, Arginine, Tomography, Optical Coherence, Diabetes Mellitus, Tipo 2, Retinopatia diabética, Coroide, Fóvea central, Óxido nítrico, Arginina, Tomografia de coerência óptica

## Abstract

**Purpose:**

To evaluate the relationship between subfoveal choroidal thickness and plasma asymmetrical dimethylarginine level and the severity of diabetic retinopathy in patients with type 2 diabetes mellitus.

**Methods:**

A total of 68 cases, including 15 patients without diabetic retinopathy, 17 patients with nonproliferative diabetic retinopathy, 16 patients with type 2 diabetes mellitus and proliferative diabetic retinopathy, and 20 healthy patients (control group), were enrolled in this study. Subfoveal choroidal thickness was measured manually using the enhanced depth imaging optical coherence tomography scanning program, and plasma asymmetrical dimethylarginine level was measured using a commercial micro enzyme-linked immunosorbent assay kit.

**Results:**

The subfoveal choroidal thickness values and plasma asymmetrical dimethylarginine levels were significantly different between the four groups (p<0.001 and p<0.001). The subfoveal choroidal thickness values were significantly lower in the proliferative diabetic retinopathy group than in the other three groups (no diabetic retinopathy, nonproliferative diabetic retinopathy, and control groups; p<0.001, p=0.045, and p<0.001, respectively). The plasma asymmetrical dimethylarginine levels were significantly higher in the proliferative diabetic retinopathy group than in the other three groups (p<0.001, p<0.04, and p<0.001, respectively). In addition, a significant negative correlation was also found between plasma asymmetrical dimethylarginine level and subfoveal choroidal thickness (p<0.001, r=-0.479).

**Conclusion:**

Asymmetrical dimethylarginine is an important marker of endothelial dysfunction and endogenous endothelial nitric oxide synthase inhibitor. The severity of diabetic retinopathy was related to increased plasma asymmetrical dimethylarginine level and reduced subfoveal choroidal thickness in type 2 diabetic patients with diabetic retinopathy.

## Abstract (pt)

**Objetivo:**

Avaliar a relação da espessura subfoveal da coroide e dos níveis plasmáticos de dimetil-arginina assimétrica com a gravidade da retinopatia diabética em pacientes com diabetes mellitus tipo 2.

**Métodos:**

Foram incluídos 68 casos, compreendendo 15 pacientes sem retinopatia diabética, 17 pacientes com retinopatia diabética não proliferativa, 16 pacientes com retinopatia diabética proliferativa, e 20 casos saudáveis (grupo de controle). A espessura subfoveal da coroide foi medida manualmente, usando o programa de varredura com tomografia computadorizada óptica com imagem profunda aprimorada, e os níveis plasmáticos de dimetil-arginina assimétrica foram medidos usando um kit microELISA comercial.

**Resultados:**

Os valores da espessura subfoveal da coroide e os níveis plasmáticos de dimetil-arginina assimétrica foram significativamente diferentes nos quatro grupos (p<0,001 para ambos os parâmetros). Os valores da espessura subfoveal da coroide foram significativamente menores no grupo com retinopatia diabética proliferativa do que nos outros três grupos (sem retinopatia diabética, retinopatia diabética não proliferativa e grupo de controle, com p<0,001, p=0,045 e p<0,001, respectivamente). Já os níveis plasmáticos de dimetil-arginina assimétrica foram significativamente maiores no grupo com retinopatia diabética proliferativa do que nos outros três grupos (p<0,001, p=0,04 e p<0,001, respectivamente). Além disso, também foi encontrada uma correlação negativa significativa entre os níveis plasmáticos de dimetil-arginina assimétrica e a espessura subfoveal da coroide (p<0,001, r=-0,479).

**Conclusão:**

A dimetil-arginina assimétrica é um importante marcador de disfunção endotelial e um inibidor endógeno da óxido nítrico sintase. Foi encontrada uma relação da gravidade da retinopatia diabética e de níveis elevados de dimetil-arginina assimétrica no plasma com a redução da espessura subfoveal da coroide em pacientes diabéticos tipo 2 com retinopatia diabética.

## INTRODUCTION

Diabetic retinopathy (DR) is one of the most common microvascular complications of diabetes. Clinical (systemic and ocular), biochemical, and molecular factors contribute to the development of DR^(1)^. DR is a progressive condition, and the factors that lead to this progression must be identified. A clinical or biochemical parameter that represents the severity of a disease can provide clinicians insights into the management of the disease, and accordingly, new treatment strategies can be developed to prevent or at least reduce the severity of complications. In recent years, many researchers have tried to find a clinical, biochemical, or molecular biomarker that reflects the severity of DR^(1)^.

Choroidal thickness and asymmetrical dimethylarginine (ADMA) level are just two of the many parameters evaluated by researchers to assess the severity of DR. The major part of ocular blood flow is choroidal circulation, whose most important function is to supply the metabolic needs of the outer retinal layers of the eye^(2)^. It is thought to contribute to changes in choroidal circulation and retinal vasculature in the development and progression of DR^(3,4)^. ADMA is an endogenous nitric oxide synthase (NOS) inhibitor responsible for the synthesis of nitric oxide (NO), which enables vascular homeostasis^(5)^. When the plasma ADMA level increases, the NO synthesis in the environment decreases, vascular homeostasis deteriorates due to vasoconstriction, and endothelial dysfunction begins^(6)^. Therefore, increased circulating ADMA level has been reported as an indicator of endothelial dysfunction^(7)^. Studies have separately investigated the relationships of severity of DR with choroidal thickness and plasma ADMA level^(4,8,9,10,11,12,13)^. However, to the best of our knowledge, no study has investigated the relationship of choroidal thickness and plasma ADMA level with the severity of DR. Accordingly, we hypothesized a relationship between plasma ADMA level and choroidal thickness and that these parameters may contribute to the progression of DR.

## METHODS

A total of 68 patients, of whom 48 had type 2 diabetes mellitus (DM) and 20 were healthy individuals, were included in this study. The study was approved by the local research ethics committee, and informed consent was obtained from all the participants in compliance with the Declaration of Helsinki on Human Rights (reference number: 2019/53).

Individuals who had a refractive error >±3 diopters and an axial length <21.5 mm and >24 mm, a history of ocular surgery, an intravitreal or subtenon injection, other ocular diseases (retinal vein occlusion, glaucoma, or ocular inflammation), a history of grid/focal or panretinal photocoagulation laser treatment, a smoking habit, hypertension, kidney failure, liver failure, heart failure, body mass index (BMI) values <18.5 and >30 kg/m^2^, and oral antidiabetic drug and insulin use were excluded from the study. After a detailed ophthalmologic examination, including assessments of refraction, axial length, best-corrected visual acuity, slit-lamp biomicroscopy, and intraocular pressure by applanation tonometer, a dilated fundus examination was performed by the same ophthalmologist. Moreover, systolic and diastolic blood pressures, BMI, and hemoglobin A1c level were examined, and the disease duration and treatments of all the patients with diabetes were recorded.

The patients with type 2 DM were classified into the following three groups in accordance with the severity of DR, with similar severity in both eyes: 1. no DR (NDR), 2. nonproliferative DR (NPDR), or 3. proliferative DR (PDR). The same clinician (U.D.) performed macular optical coherence tomography (OCT) for all the participants using a SPECTRALIS OCT system (SPECTRALIS HRA+OCT; Heidelberg Engineering, Heidelberg, Germany). Only high-quality images were accepted. All the measurements were performed before noon to prevent diurnal variations in choroidal thickness. The subfoveal choroidal thickness (SCT) was measured manually by a different clinician (M.C.) who was blinded to the groups by using an enhanced depth imaging OCT (EDI-OCT) program.

Blood samples were collected from all the participants into vacuum EDTA (ethylenediaminetetraacetic acid) tubes for ADMA measurement and centrifuged at +4 for 5 minutes. The plasma samples were stored at -80°C until analysis. Plasma ADMA levels were measured using an ADMA micro enzyme-linked immunosorbent assay kit (Immune Diagnostics, Inc.).

### Statistical analyses

Statistical analyses were performed using SPSS 20.0 for Windows (SPSS Inc., Chicago, Illinois, USA). For the measurement of SCT, only data from the right eye were used in the statistical analysis. The normality of data was analyzed with the Shapiro-Wilk test.

Descriptive statistics are presented as mean ± standard deviation. Multiple comparisons between groups were performed using a one-way analysis of variance. The Bonferroni post hoc test was used for pairwise comparisons of the groups. The Pearson correlation coefficient test was used for the correlation analysis. A p value < 0.05 is considered statistically significant.

## RESULTS

The demographic, ocular, and systemic characteristics of the groups are summarized in table 1. The groups were similar in terms of age and sex (p=0.484 and p=0.997, respectively). The mean SCT values were 300.4 ± 20.6, 267.35 ± 34.54, 238 ± 38.66, and 302.6 ± 25.13 µm in the NDR, NPDR, PDR, and control groups, respectively (p<0.001). The Bonferroni post hoc test revealed that the mean SCT values in the PDR group were lower than those in the NDR, NPDR, and control groups (p<0.001, p=0.045, and p<0.001, respectively; Table 2). In addition, the values in the NPDR group were statistically significantly lower than those in the NDR and control groups (p=0.019 and p=0.005, respectively).

**Table 1.** Demographic, ocular, and systemic characteristics of the groups

Variable	NDR (n=15)	NPDR (n=17)	PDR (n=16)	Control (n=20)
Age (years)	53.26 ± 9.02	51.29 ± 9.31	56.37 ± 10.72	54.55 ± 9.26
Sex (female/male)	7F/8M	8F/9M	7F/9M	9F/11M
BCVA (Snellen)	0.94 ± 0.09	0.68 ± 0.22	0.17 ± 0.11	0.97 ± 0.06
IOP (mmHg)	15.8 ± 1.69	15.23 ± 2.46	15.06 ± 2.08	16.05 ± 1.76
AL (mm)	22.61 ± 0.61	22.90 ± 0.72	22.59 ± 0.60	22.61 ± 0.61
DBP (mmHg)	80.8 ± 7.77	80.23 ± 5.10	80.68 ± 8.03	78.45 ± 7.55
SBP (mmHg)	119.2 ± 11.05	121.11 ± 8.46	122.6 ± 11.10	117.95 ± 10.02
BMI (kg/m^2^	26.2 ± 2.15	25.95 ± 2.07	26.87 ± 1.78	25.39 ± 2.02
HbA1c(mmol/mol)	7.47 ± 1.52	8.68 ± 1.57	9.43 ± 1.33	4.96 ± 0.27
Duration of DM (years)	10.4 ± 3.13	11.52 ± 3.67	13.93 ± 4.83	-
Medical treatment	WMT = 2	WMT = 4	WMT = 1	-
	OAD = 8	OAD = 9	OAD = 3	
	Insulin - 5	Insulin = 4	Insulin = 12	

NDR= No diabetic retinopathy; NPDR= Nonproliferative diabetic retinopathy; PDR= Proliferative diabetic retinopathy; BCVA= Best-corrected visual acuity; IOP= Intraocular pressure; AL= Axial length; DBP= Diastolic blood pressure; SBP= Systolic blood pressure; BMI: Body mass index; HbA1c:= Hemoglobin A1c DM= Diabetus mellitus; WMT= Without medical treatment; OAD= Oral antidiabetic.

**Table 2.** ADMA levels and SCT values of the groups

Variable	NDR (n=15)	NPDR (n=17)	PDR (n=16)	Control (n=20)	p*	Post hoc test
**SCT (µm)**	300.4 ± 20.6	267.35 ± 34.54	238 ± 38.66	302.6 ± 25.13	<0.001	**PDR-NDR < 0.001** **PDR-NPDR = 0.045** **PDR-Control: < 0.001** **NPDR-NDR = 0.019** **NPDR-Control = 0.005**
**ADMA (µmol/L)**	0.25 ± 0.13	0.41 ± 0.15	0.55 ± 0.16	0.27 ± 0.13	<0.001	**PDR-NDR < 0.001** **PDR-NPDR = 0.04** **PDR-Control < 0.001** **NPDR-NDR = 0.018** **NPDR-Control = 0.029**

NDR= No diabetic retinopathy; NPDR= Nonproliferative diabetic retinopathy; PDR= Proliferative diabetic retinopathy; SCT= Subfoveal choroidal thickness; ADMA= Plasma asymmetrical dimethylarginine.

*Obtained using analysis of variance.

The plasma ADMA levels were 0.25 ± 0.13, 0.41 ± 0.15, 0.55 ± 0.16, and 0.27 ± 0.13 µmol/L in the NDR, NPDR, PDR, and control groups, respectively (p<0.001). The Bonferroni post hoc test revealed that the ADMA level was significantly higher in the PDR group than in the NDR, NPDR, and control groups (p<0.001, p=0.04, and p<0.001, respectively; Table 2). The mean values in the NPDR group were also significantly higher than those in the NDR and control groups (p=0.018 and p=0.029, respectively). A significant severe negative correlation was also found between the plasma ADMA level and SCT (p<0.001, r=-0.479).

## DISCUSSION

DR is one of the leading causes of blindness. Many factors, particularly the duration of diabetes, adversely affect the development and progression of DR. In recent years, interest has been increasing in identifying the factors that contribute to the development and progression of DR and to elucidate their mechanisms.

Certainly, the choroid, which has a rich vascular network, is affected by vascular complications due to diabetes. In postmortem studies in patients with diabetes, pathological vascular changes, including neovascularization, capillary atrophy, capillary narrowing, and endothelial destruction, have been observed in the choroid^(14)^. In vivo conditions, indocyanine green angiography, laser Doppler flowmetry, ultrasonography, and EDI-OCT are the most commonly used methods for evaluating the choroid^(15,16,17)^. Although all of these techniques allow the detection of choroidal vessel abnormalities and changes in blood flow, only EDI-OCT could obtain in vivo cross-sectional images that provide information on the true choroidal thickness and morphology, which help distinguish normal and pathological processes in the choroid. Choroidal thickness measurements obtained with EDI-OCT, which is highly reproducible and reliable, have helped us to elucidate the etiopathogenesis of some diseases, evaluate their severity, treatment efficacy, and follow-up recurrences^(8,9,10,11,18,19)^.

Many studies have been performed to understand the relationship between DR and choroidal angiopathy. The relationship between choroidal thickness and the severity of DR was also investigated. Most studies have reported the thinning of the choroidal thickness in patients with diabetic eye disease. However, the existence of a relationship between the severity of DR and choroidal thickness is unclear because in some studies, choroidal thinning has been observed in diabetic eyes but has not shown any correlation with the severity of DR^(20,21,22)^. By contrast, some studies have reported a negative correlation with the severity of DR, while others have reported a positive correlation^(4,23,24)^. Campos et al^(20)^ reported that the discreoancy in the results of studies that examined the relationship between choroidal thickness and ADMA level may be related to some factors that may affect choroidal thickness, such as sample size, age range of the study subjects, application of intravitreal injection or laser photocoagulation therapy, the type of OCT procedure used, and the presence of other systemic diseases.

In our study, we formed study groups by paying attention to the aforementioned factors as much as possible, and our results show that choroidal thinning occurs in diabetic eyes, similar to the findings of other studies. However, as the severity of DRP increases, the choroidal thickness significantly decreases. The choroid consists of five layers, namely the suprachoroid lamina (with expansion lacunae), Haller’s vessel layer, Sattler’s vessel layer, choriocapillaris, and Bruch’s membrane. Nickla and Wallman^(2)^ suggested that lacunae in the suprachoroid layer may be responsible for the changes in choroidal thickness. According to the authors, changes in the tone of nonvascular smooth muscle cells, which are particularly found under the fovea, can affect the choroidal thickness by changing the size of the lacunae^(2)^. The authors suggested that NOS was found in the positive axon terminals of the nonvascular smooth muscle cells in the choroid and that NO synthesized by NOS is a possible mechanism that can increase the choroidal thickness by loosening the smooth muscles and causing expansion of the lacunae^(2)^.

However, NOS is expressed not only in these smooth muscle cells but also in the ganglion cell plexus tans of the choroid consisting of large vessels^(25)^. The choroidal vascular tone also affects the choroidal thickness^(2)^. NOS, which is responsible for the release of NO, affects the width of lacunae and vessels. Thus, it may be one of the factors that affect choroidal thickness.

Accordingly, we hypothesized that the endogenous NOS inhibitor ADMA may be one of the factors that cause the choroidal thinning in patients with diabetes. By measuring the plasma ADMA levels of the participants, we investigated the relationship between the severity of DR and choroidal thickness.

Just like choroidal thickness, the relationship between plasma ADMA level and DR has been highly studied by researchers in recent years. ADMA has been described as an indicator of endothelial dysfunction^(7)^. Diabetes is a disease with microvascular and macrovascular complications. Endothelial dysfunction is the earliest sign of vascular complications. DR is one of the microvascular complications of diabetes, and as in the case of choroidal thickness, plasma ADMA levels have been found to generally increase in many studies, particularly in the PDR group^(12,13,26,27,28)^. Although the plasma ADMA level inhibits vascular endothelial growth factor-mediated angiogenesis, it also contributes to angiogenesis by increasing ephrin-B2 expression, an important angiogenesis factor in diabetes^(29)^.

Similarly with other studies, we found that plasma ADMA levels increased in the patients with DR, and this increase was directly proportional to the severity of diabetes. However, to evaluate our hypothesis that increased plasma ADMA level may contribute to the progression of DR by affecting the choroidal thickness, we examined the relationship between plasma ADMA level and choroidal thickness. On the basis of our results, choroidal thickness significantly decreased as the plasma ADMA level increased. This study was not the first to evaluate the relationship between plasma ADMA level and choroidal thickness.

Balmforth et al. examined the relationship between choroidal thickness and plasma ADMA level in patients with chronic kidney disease and reported a negative correlation^(30)^. However, in their study, they excluded patients with diabetes. In this respect, our study is the first to investigate the relationship of plasma ADMA level and choroidal thickness with the severity of DR in patients with diabetes.

However, our study has several limitations. First, our sample size was small because we excluded many factors that could affect choroidal thickness and plasma ADMA levels. Second, our study lacks simultaneous evaluations of plasma ADMA and aqueous ADMA level, which could have made our study more valuable.

Choroidal thickness is a clinical marker, and plasma ADMA level is a biochemical marker that may reflect the vascular effects of diabetes, and these markers are related to each other. Our results suggest that the relationship between the two parameters may have an impact on the development and progression of diabetes. Plasma ADMA levels in patients with diabetes may be a new therapeutic target. Keeping the choroidal blood flow within normal limits may prevent the development or rapid progression of DR. To better understand the role of the relationship between choroidal thickness and plasma ADMA levels in the development and progression of DR, studies that involve larger patient populations are needed.

## References

[1] Jenkins AJ, Joglekar MV, Hardikar AA, Keech AC, O’Neal DN, Januszewski AS (2015). Biomarkers in diabetic retinopathy. Rev Diabet Stud.

[2] Nickla DL, Wallman J (2010). The multifunctional choroid. Prog Retin Eye Res.

[3] Hua R, Liu L, Wang X, Chen L (2013). Imaging evidence of diabetic choroidopathy in vivo: angiographic
pathoanatomy and choroidal-enhanced depth imaging. PLoS One.

[4] Laíns I, Talcott KE, Santos AR, Marques JH, Gil P, Gil J (2018). Choroidal thickness in diabetic retinopathy assessed with
swept-source optical coherence tomography. Retina.

[5] Leiper JM, Vallance P (2006). The synthesis and metabolism of asymmetric dimethylarginine
(ADMA). Eur J Clin Pharmacol.

[6] Endemann DH, Schiffrin E (2004). Endothelial dysfunction. J Am Soc Nephrol.

[7] Cooke JP (2000). Does ADMA cause endothelial dysfunction?. Arterioscler Thromb Vasc Biol.

[8] Regatieri CV, Branchini L, Carmody J, Fujimoto JG, Duker JS (2012). Choroidal thickness in patients with diabetic retinopathy
analyzed by spectral-domain optical coherence tomography. Retina.

[9] Wang H, Tao Y (2019). Choroidal structural changes correlate with severity of diabetic
retinopathy in diabetes mellitus. BMC Ophthalmol.

[10] Rewbury R, Want A, Varughese R, Chong V (2016). Subfoveal choroidal thickness in patients with diabetic
retinopathy and diabetic macular oedema. Eye (Lond).

[11] Kim JT, Lee DH, Joe SG, Kim JG, Yoon YH (2013). Changes in choroidal thickness in relation to the severity of
retinopathy and macular edema in type 2 diabetic patients. Invest Ophthalmol Vis Sci.

[12] Abhary S, Kasmeridis N, Burdon KP, Kuot A, Whiting MJ, Yew WP (2009). Diabetic retinopathy is associated with elevated serum asymmetric
and symmetric dimethylarginines. Diabetes Care.

[13] Malecki MT, Undas A, Cyganek K, Mirkiewicz-Sieradzka B, Wolkow P, Osmenda G (2007). Plasma asymmetric dimethylarginine (ADMA) is associated with
retinopathy in type 2 diabetes. Diabetes Care.

[14] Hidayat AA, Fine BS (1985). Diabetic choroidopathy. Light and electron microscopic
observations of seven cases. Ophthalmology.

[15] Gugleta K, Orgül S, Flammer I, Gherghel D, Flammer J (2002). Reliability of confocal choroidal laser Doppler
flowmetry. Invest Ophthalmol Vis Sci.

[16] Spaide RF, Yannuzzi LA, Slakter JS, Sorenson J, Orlach DA (1995). Indocyanine green videoangiography of idiopathic polypoidal
choroidal vasculopathy. Retina.

[17] Spaide RF, Koizumi H, Pozzoni MC (2008). Enhanced depth imaging spectral-domain optical coherence
tomography. Am J Ophthalmol.

[18] Fujiwara T, Imamura Y, Margolis R, Slakter JS, Spaide RF (2009). Enhanced depth imaging optical coherence tomography of the
choroid in highly myopic eyes. Am J Ophthalmol.

[19] Imamura Y, Fujiwara T, Margolis R, Spaide RF (2009). Enhanced depth imaging optical coherence tomography of the
choroid in central serous chorioretinopathy. Retina.

[20] Campos A, Campos EJ, Martins J, Ambrósio AF, Silva R (2017). Viewing the choroid: where we stand, challenges and
contradictions in diabetic retinopathy and diabetic macular
oedema. Acta Ophthalmol.

[21] Esmaeelpour M, Brunner S, Ansari-Shahrezaei S, Nemetz S, Povazay B, Kajic V (2012). Choroidal thinning in diabetes type 1 detected by 3-dimensional
1060 nm optical coherence tomography. Invest Ophthalmol Vis Sci.

[22] Querques G, Lattanzio R, Querques L, Del Turco C, Forte R, Pierro L (2012). Enhanced depth imaging optical coherence tomography in type 2
diabetes. Invest Ophthalmol Vis Sci.

[23] Lee HK, Lim JW, Shin MC (2013). Comparison of choroidal thickness in patients with diabetes by
spectral-domain optical coherence tomography. Korean J Ophthalmol.

[24] Kim JT, Lee DH, Joe SG, Kim JG, Yoon YH (2013). Changes in choroidal thickness in relation to the severity of
retinopathy and macular edema in type 2 diabetic patients. Invest Ophthalmol Vis Sci.

[25] Flugel C, Tamm ER, Mayer B, Lutjen-Drecoll E (1994). Species differences in choroidal vasodilative innervation:
evidence for specific intrinsic nitrergic and VIP-positive neurons in the
human eye. Invest Ophthalmol Vis Sci.

[26] Krzyzanowska K, Mittermayer F, Schernthaner GH, Brunner S, Brix JM, Aschauer S (2011). Renal function but not asymmetric dimethylarginine is
independently associated with retinopathy in type 2 diabetes. Cardiol Res Pract.

[27] Tarnow L, Hovind P, Teerlink T, Stehouwer CD, Parving HH (2004). Elevated plasma asymmetric dimethylarginine as a marker of
cardiovascular morbidity in early diabetic nephropathy in type 1
diabetes. Diabetes Care.

[28] Yonem A, Duran C, Unal M, Ipcioglu OM, Ozcan O (2009). Plasma apelin and asymmetric dimethylarginine levels in type 2
diabetic patients with diabetic retinopathy. Diabetes Res Clin Pract.

[29] Du MR, Yan L, Li NS, Wang YJ, Zhou T, Jiang JL (2018). Asymmetric dimethylarginine contributes to retinal
neovascularization of diabetic retinopathy through EphrinB2
pathway. Vascul Pharmacol.

[30] Balmforth C, van Bragt JJ, Ruijs T, Cameron JR, Kimmitt R, Moorhouse R (2016). Chorioretinal thinning in chronic kidney disease links to
inflammation and endothelial dysfunction. JCI Insight.

